# Everybody's Page

**Published:** 1891-08-01

**Authors:** 


### Everybody's Page.

Dr. Tarnier is stated, in a German medical journal, to have been using for some time with much success in obstetrical practice a five per cent. solution of sulphate of copper. He claims for it, that it is an unusually active disinfectant, relatively non-toxic, and very soluble. It has the additional advantage of being cheap and readily obtainable.

Dr. Hertel claims for a substance called "hydrochlorate of phenocoll," that it has both most useful antipyretic properties and specific action in articular rheumatism. The advantages claimed for the compound are, that it exercises no hurtful influence on the blood, in contrast to most antipyretics, and is practically non-toxic to animals.

The millennium cannot be far off. Some enterprising gentle men in Kansas City are reported to be forming a company, the object of which is to pump hot air into the city during the winter months and cold air during the summer! It only remains for these benevolently-disposed persons to form a company for the supply gratis of unlimited means to poor people, and of appetites and some pleasurable emotions to the wealthy, in order to earn the eternal gratitude of the human race.

Dr. Neovius writes enthusiastically, in a Finnish journal, on the properties of common thyme in combating whooping-cough. His observations lead him to the conclusion, that if given *fresh*, in doses of from one ounce and a-half to six ounces a day mixed with a little marsh-mallow syrup, regularly for at least two weeks, it is practically a specific. Given at once, the symptoms vanish in two or three days, and in a fortnight the disease is expelled. Its simplicity and comparative harmlessness, combined with the fact that it is a remedy within the means of the poorest, is a strong recommendation.

A curious and unpleasant case of explosion is reported in *La Pratique Médicale*. A patient had had compressed tablets of potassium chlorate prescribed for him, and carried them about in his pocket wrapped up in a piece of paper. Judging by what happened, these tablets are as unmannerly in their behaviour, and as much to be avoided, as Whitehead torpedoes. One day on sitting down he was startled by an explosion which was followed immediately by a scorching of his body over an extent of fully nine inches. The tablets, it appears, had come in contact with a penknife, to whose intrusion they evidently objected, but they showed unnecessary warmth—so the patient thought.

A somewhat novel treatment of phthisis was expounded at a recent meeting of the Académie de Médecine by Dr. Germain Sée. His method of arresting the progress of the disease, by which he considers he has practically cured twelve cases, is to place the patient for two or three hours every day in a compartment filled with compressed air containing the vapours of creosote mixed with eucalyptus. It is the combination of these which has such a favourable influence on the patient. A very large amount of creosote is thus absorbed by the entire pulmonary surface, and it can be supported where a very small dose administered internally cannot be continued for any length of time. Under its influence, when inhaled in this manner, Dr. Sée, even in the most advanced cases of phthisis, has seen the appetite return and improvement of general symptoms.

The Legislature of North Carolina has set an example which might be followed with advantage by other states and counties. A portion of the Western North Carolina Hospital has been set aside for the care and treatment of inebriates.

The necessity for making the bill of fare attractive to invalids is apparently so often forgotten, that those who know the sufferings of a sick bed will be grateful to Dr. R. W. Burnet for laying stress upon this fact in his excellent manual on "Foods and Dietaries."

In all things the Americans are practical. They have shown this decisively in their settlement of the question of the contagiousness of leprosy. In the last annual report of the Marine Hospital Service of the United States, Supervising Surgeon-General Hamilton remarks: "There may be some question whether leprosy is contagious, but there can be none that lepers are undesirable additions to our population." Acting upon this sensible view of the case, it has been decided not to admit lepers into the territory of the United States.

Mr. Montagu Williams has an affection for the "cat." Not pussy, so dear to the heart of the maiden lady in her solitude, but the lash. He does not stand alone in the opinion that what proved the grave of garotting is the only likely punishment to put down the brutality of those husbands who beat and ill-use their wives. But what a clamour would arise from the shrieking brotherhood and sisterhood on the introduction of the "cat." They bleat idiotically about its brutalising effects, but how can you brutalise what is already lower than the so-called brutes?

The value of Dr. Wallace's evidence before the Royal Commission on Vaccination is not enhanced by the admission on his part that his tables, on the accuracy of which so much depended, were "not perfect." It is still further decreased by his withdrawing the remarkable assertion that "the amount of small-pox mortality increases as the amount of vaccination increases," and confessing, after the inaccuracy of his tables was pointed out, that he did not wish to put this dictum forward as proved by his diagram. If there is anything in the opinions of Dr. Wallace and the anti-vaccinators whose cause he is championing, it would have been wiser of him to have taken up the position of pure science rather than of haphazard assertion.

M. de Brun, Professor of Medicine at Beyrout, has been observing and comparing epidemics of dengue fever with the recent outbreaks of influenza, with results which point to the fact that they are not one and the same disease. Out of two thousand cases of dengue fever which he has observed during the past three years in Syria, he has not observed one in which there were severe pulmonary symptoms which are so characteristic of influenza. It is the gastric symptoms which he has noticed that differentiate dengue from the latter disease, and the frequent presence of a bright red erythema in the early stage. The points of similarity appear to be the pains in the head, back, and legs, and the irregularity of the temperature. M. de Brun's experience as to the absence of pulmonary trouble in dengue is confirmed by that of other observers in districts bordering on the Mediterranean.

